# Optimal Central Obesity Measurement Site for Assessing Cardiometabolic and Type 2 Diabetes Risk in Middle-Aged Adults

**DOI:** 10.1371/journal.pone.0129088

**Published:** 2015-06-04

**Authors:** Seán R. Millar, Ivan J. Perry, Jan Van den Broeck, Catherine M. Phillips

**Affiliations:** 1 HRB Centre for Health and Diet Research, Department of Epidemiology and Public Health, University College Cork, Cork, Ireland; 2 Department of Global Public Health and Primary Care, University of Bergen, Bergen, Norway; University of Leipzig, GERMANY

## Abstract

**Objectives:**

Despite recommendations that central obesity assessment should be employed as a marker of cardiometabolic health, no consensus exists regarding measurement protocol. This study examined a range of anthropometric variables and their relationships with cardiometabolic features and type 2 diabetes in order to ascertain whether measurement site influences discriminatory accuracy. In particular, we compared waist circumference (WC) measured at two sites: (1) immediately below the lowest rib (WC rib) and (2) between the lowest rib and iliac crest (WC midway), which has been recommended by the World Health Organisation and International Diabetes Federation.

**Materials and Methods:**

This was a cross-sectional study involving a random sample of 2,002 men and women aged 46-73 years. Metabolic profiles and WC, hip circumference, pelvic width and body mass index (BMI) were determined. Correlation, logistic regression and area under the receiver operating characteristic curve analyses were used to evaluate obesity measurement relationships with metabolic risk phenotypes and type 2 diabetes.

**Results:**

WC rib measures displayed the strongest associations with non-optimal lipid and lipoprotein levels, high blood pressure, insulin resistance, impaired fasting glucose, a clustering of metabolic risk features and type 2 diabetes, in both genders. Rib-derived indices improved discrimination of type 2 diabetes by 3-7% compared to BMI and 2-6% compared to WC midway (in men) and 5-7% compared to BMI and 4-6% compared to WC midway (in women). A prediction model including BMI and central obesity displayed a significantly higher area under the curve for WC rib (0.78, P=0.003), Rib/height ratio (0.80, P<0.001), Rib/pelvis ratio (0.79, P<0.001), but not for WC midway (0.75, P=0.127), when compared to one with BMI alone (0.74).

**Conclusions:**

WC rib is easier to assess and our data suggest that it is a better method for determining obesity-related cardiometabolic risk than WC midway. The clinical utility of rib-derived indices, or alternative WC measurements, deserves further investigation.

## Introduction

Obesity is associated with dyslipidaemia, hypertension, insulin resistance and the development of metabolic syndrome and type 2 diabetes [1], leading to a greater likelihood of premature death. However, not all obese subjects are at increased cardiometabolic risk as a proportion are considered to be metabolically healthy [2]. The prevalence of obesity has escalated in many world populations [3]. Thus, there is an increasing need for inexpensive and non-invasive methods for use in clinical practice to identify overweight and obese individuals at highest odds of developing metabolic abnormalities and type 2 diabetes.

Body mass index (BMI) has traditionally been the chosen surrogate method used to determine excess body fat, but because it is a weight-for-height measure, BMI is unable to distinguish between fat and lean mass. Recent research has indicated that general obesity categorisation based on BMI might be inadequate [4,5], and studies have shown that BMI may misclassify adiposity [6–8].

Increasing evidence suggests that central obesity is a more important cardiometabolic risk factor [9,10] and waist circumference (WC) measurement has been recommended as a method for central obesity assessment. However, partly due to a lack of agreement on a universal measurement protocol, its clinical usefulness and superiority over BMI in the prediction of cardiometabolic events has been questioned [11,12]. Various transformations of WC have also been used, such as the waist/height ratio (WHtR) [13] and waist/hip ratio (WHR) [14]. Although extensive research has attempted to quantify relationships between different adiposity measures and morbidity [11], considerable controversy still exists as to which measurement site or index most accurately defines non-optimal body fat distribution [15].

In this study we examined a range of anthropometric variables and their relationships with metabolic risk phenotypes, including lipid and lipoprotein levels, high blood pressure, insulin resistance, impaired fasting glucose, a clustering of metabolic risk features and type 2 diabetes, in a random sample of 2,002 middle-aged men and women. In particular, we compared the discriminatory performance of WC measured at two locations (immediately below the lowest rib, and between the lowest rib and iliac crest), and variations of these measures, to address the hypothesis that the measurement site for central obesity affects its accuracy as a predictor of cardiometabolic risk.

## Materials and Methods

### Study population

The Cork and Kerry Diabetes and Heart Disease Study (Phase II) was a cross-sectional study conducted between 2010 and 2011. A random sample was recruited from a large primary care centre in Mitchelstown, County Cork, Ireland. The Livinghealth Clinic serves a population of approximately 20,000, with a mix of urban and rural residents. Stratified sampling by age and sex was employed to recruit equal numbers of men and women from all registered attending patients in the 50–69 year age group. In total, 3,807 individuals were selected from the practice list. Following the exclusion of duplicates, deaths, and subjects incapable of consenting or attending appointment, 3,051 were invited to participate in the study and of these, 2,047 (49.2% male) completed the questionnaire and physical examination components of the baseline assessment (response rate: 67.1%). Details regarding the study design, sampling procedures and methods of data collection have been reported previously [16].

Ethics committee approval conforming to the Declaration of Helsinki was obtained from the Clinical Research Ethics Committee of University College Cork. A letter signed by the contact GP in the clinic was sent out to all selected participants with a reply slip indicating acceptance or refusal. All subjects gave signed informed consent, including permission to use their data for research purposes.

### Clinical and laboratory measurements

All study participants attended the clinic in the morning after an overnight fast and blood samples were taken on arrival. Data on age, gender, physician-diagnosed type 2 diabetes and prescription (Rx) medication use were gathered through a self-completed General Health Questionnaire. Triglyceride and high density lipoprotein cholesterol (HDL-C) levels were measured by Cork University Hospital Biochemistry Laboratory on Olympus 5400 biochemistry analysers with Olympus reagents using standardised procedures and fresh samples (Olympus Diagnostica GmbH, Hamburg, Germany). Fasting plasma glucose concentrations were determined using a glucose hexokinase assay (Olympus Life and Material Science Europa Ltd., Lismeehan, Co. Clare, Ireland) and fasting serum insulin was calculated using a biochip array system (Evidence Investigator; Randox Laboratories, UK). Glycated haemoglobin A_1c_ (HbA_1c_) levels were measured in the haematology laboratory on an automated high-pressure liquid chromatography instrument Tosoh G7 [Tosoh HLC-723 (G7), Tosoh Europe N.V, Tessenderlo, Belgium]. Three independent measurements of systolic and diastolic blood pressure (BP) were obtained with the subject in a seated position using an Omron M7 digital sphygmomanometer (Omron Healthcare Co. Ltd., Japan). The mean of the second and third readings was considered to be a subject’s BP.

### Anthropometric variables

Anthropometric measurements were taken by researchers who were thoroughly trained according to the study research protocols [16]. The weight and height of each subject were measured to the nearest 0.1 kg and 0.1 cm respectively. Portable electronic Tanita WB-100MA weighing scales (Tanita Corporation, IL, USA) were placed on a firm, flat surface and were calibrated weekly to ensure accuracy. Height was assessed using a portable Seca Leicester height/length stadiometer (Seca, Birmingham, UK) and BMI was calculated as weight divided by the square of height. Midway WC (WC midway) was measured between the lowest rib and iliac crest on bare skin. Participants were instructed to breathe in, and then out, and to hold their breath while measurement was made to the nearest 0.1 cm using a Seca 200 measuring tape. Rib WC (WC rib) was measured immediately below the lowest rib at the mid-axillary line and hip circumference was determined at the maximum perimeter of the hips. Pelvic width was calculated as the diameter between the right and left iliac crests using callipers. For each central obesity measure, the mean of two independent readings was used in analysis. Height, hip circumference and pelvic width were divided into WC midway and WC rib measurements deriving six variables: (1) *Midway/height ratio*, (2) *Midway/hip ratio*, (3) *Midway/pelvis ratio* and (4) *Rib/height ratio*, (5) *Rib/hip ratio*, (6) *Rib/pelvis ratio*.

### Classification of biochemical and blood pressure measurements

Lipid, lipoprotein, glucose and BP measurements were categorised according to National Cholesterol Education Program Adult Treatment Panel III criteria [17]. Abnormal metabolic risks were defined as high triglyceride levels ≥1.7 mmol/l, low HDL-C (<1.03 mmol/l in males or <1.29 mmol/l in females) and impaired fasting glucose levels 5.6–6.9 mmol/l. High BP was classified as systolic BP ≥130 mmHg and/or diastolic BP ≥85 mmHg or Rx anti-hypertensive medication use. The Homeostasis Model Assessment Index of Insulin Resistance (HOMA-IR) [18] was derived from fasting glucose and insulin concentrations as [(fasting plasma glucose x fasting serum insulin)/22.5], and insulin resistance was defined as a level equal to or above the 75^th^ percentile in the study population. Having three or more cardiometabolic risk features was characterised as any combination of these variables. According to American Diabetes Association guidelines, type 2 diabetes was defined as HbA_1c_ ≥6.5% (≥48 mmol/mol) or fasting plasma glucose ≥7.0 mmol/l [19]. Individuals on insulin therapy and subjects indicating a diagnosis of diabetes (either self-reported physician diagnosis or Rx diabetes medication use), but who did not have positive HbA_1c_ or fasting plasma glucose test results, were excluded from analysis (N = 45).

### Statistical analysis

The distribution of each metabolic characteristic was assessed using Shapiro-Wilk and Kolmogorov-Smirnov statistics. Categorical features are presented as percentages and continuous data are shown as a mean, plus or minus one standard deviation, or a median and interquartile range. Gender differences were evaluated using chi-square tests, independent *t*-tests or a Mann-Whitney U for skewed data. Relationships between anthropometric measurements and continuous cardiometabolic variables were investigated using partial correlations. Variables presenting a non-normal distribution were log-transformed. All obesity measures were gender-standardised and separate and stratified binary logistic regression models were used to compare index associations with cardiometabolic risk features and type 2 diabetes, adjusting for age.

The ability of selected indices to discriminate three or more cardiometabolic risk features and type 2 diabetes was measured using receiver operating characteristic curve (ROC) analysis. The area under the curve (AUC) provides a scale from 0.5 to 1.0 (with 0.5 representing random chance and 1.0 indicating perfect discrimination) by which to appraise the capacity of an obesity index to detect a positive result [20]. A higher AUC generally indicates greater diagnostic accuracy. Covariate-adjusted analysis [21] was performed to account for the potential confounding influence of both age and gender (full cohort) or age alone in stratified models. The AUC values were compared for statistical differences and were further evaluated by determining false positive rates at specific points on the curve corresponding to 90%, 80%, 70% and 60% sensitivities.

To further judge the ability of central obesity to discriminate type 2 diabetes, we compared a logistic regression prediction model containing BMI to models which included both BMI and selected central obesity measures. The accuracy of each model was assessed using the ROC curve. We additionally evaluated discrimination using Integrated Discrimination Improvement (IDI) analysis, which indicates the magnitude of improvement in the performance of a model by adding another variable [22]. To assess goodness-of-fit, the likelihood ratio (LR) chi-square statistics were examined by comparing models with or without an additional anthropometric measure. Calibration was measured using the Hosmer-Lemeshow (HL) test.

Data analysis was conducted using IBM SPSS Statistics Version 20 (IBM Corp., Armonk, NY, USA) and Stata SE Version 13 (Stata Corporation, College Station, TX, USA) for Windows. Seven subjects had missing anthropometric values. For all analyses, a P value (two-tailed) of less than 0.05 was considered to indicate statistical significance.

## Results

### Descriptive characteristics

Characteristics of the study population are presented in Table 1. According to BMI classification recommended by the World Health Organisation (WHO) [23], 1,550 (77.7%) participants were either overweight or obese, with 835 (85.6%) male subjects having a BMI ≥25 kg/m^2^ compared to 715 (70.2%) females (P for difference <0.001). Mean WC and pelvic width measurements were also significantly increased in men while hip circumference levels were greater in women. Distinctions between WC midway and WC rib were observed in both genders, with average midway values being higher. With consideration to metabolic risk factors, male subjects were significantly more likely to have abnormal triglyceride levels, high BP, insulin resistance, impaired fasting glucose, a clustering of cardiometabolic risk features and type 2 diabetes.

**Table 1:** Characteristics of the study population.

Feature	Males	Females	P value
	(N = 981)	(N = 1021)	
Age	59 (55–64)	59.0 (54–64)	0.791
Weight (kg)	87.38 ± 13.8	71.58 ± 13.6	<0.001
Height (m)	1.73 ± 0.1	1.60 ± 0.1	<0.001
BMI (kg/m2)	29.12 ± 4.2	28.02 ± 5.2	<0.001
WC midway (cm)	102.61 ± 11.1	91.37 ± 12.7	<0.001
WC rib (cm)	99.88 ± 10.1	85.10 ± 12.2	<0.001
Hip circumference (cm)	98.96 ± 8.7	101.79 ± 10.7	<0.001
Pelvic width (cm)	32.96 ± 2.4	31.97 ± 2.7	<0.001
Triglycerides (mmol/l)	1.32 (0.9–1.9)	1.10 (0.8–1.5)	<0.001
High triglycerides^1^	313 (32.9)	164 (16.5)	<0.001
HDL-C (mmol/l)	1.28 ± 0.3	1.62 ± 0.4	<0.001
Low HDL-C^2^	166 (17.3)	169 (16.8)	0.676
Average systolic BP (mmHg)	130.83 ± 15.6	128.44 ±17.9	0.001
Average diastolic BP (mmHg)	79.94 ± 9.6	80.42 ± 9.9	0.339
High blood pressure^3^	628 (64.3)	593 (58.3)	0.006
HOMA-IR	3.27 (1.3–3.8)	2.32 (1.0–2.7)	<0.001
Insulin resistance^4^	301 (32.0)	179 (18.2)	<0.001
Fasting plasma glucose (mmol/l)^5^	5.00 (4.7–5.4)	4.80 (4.5–5.2)	<0.001
Impaired fasting glucose^5^ ^,^ ^6^	150 (17.3)	80 (8.5)	<0.001
Three or more cardiometabolic risk features^5^	178 (20.0)	106 (10.9)	<0.001
Type 2 diabetes	92 (9.5)	50 (5.0)	<0.001

Mean and ± standard deviation are shown for continuous variables, P value calculated with a Student’s *t*-test. Age, triglycerides, HOMA-IR, HbA_1c_ and fasting plasma glucose are shown as a median (interquartile range) with a P value according to a Mann-Whitney U. % are shown for categorical values with *x* ^2^ for difference in proportions, numbers and (%) may vary as some variables have missing values.

^1^Triglycerides ≥1.7 mmol/l.

^2^HDL-C <1.03 mmol/l (males) or HDL-C <1.29 mmol/l (females).

^3^BP ≥130/85 mmHg or on Rx for hypertension.

^4^HOMA-IR 75^th^ percentile.

^5^Excluding subjects with type 2 diabetes.

^6^Fasting plasma glucose ≥5.6 mmol/l.

### Partial correlations between anthropometric measurements and cardiometabolic variables

After adjustment for age, positive correlations for triglycerides, systolic BP, diastolic BP, HbA_1c_, glucose, HOMA-IR, and negative correlations for HDL-C, were observed with weight, BMI and measurements of central obesity (Table 2). Significant inverse relationships were also noted for height with triglyceride and glucose concentrations in men, while HDL-C was positively correlated with height in women. Relationships were stronger between WC rib and a majority of metabolic variables, with triglycerides, HDL-C and HOMA-IR showing the highest correlative strengths. Nevertheless, metabolic variable correlations with BMI and WC midway, although reduced, were of a similar magnitude in men.

**Table 2:** Partial correlations^1^ between anthropometric measurements and cardiometabolic variables, stratified by gender.

Cardiometabolic feature	Weight	Height	BMI	WC midway	WC rib	Hip circumference	Pelvic width
MALES							
Triglycerides^2^	0.249	-0.062	0.306	0.296	**0.319**	0.257	0.162
HDL-C	-0.347	0.063^3^	-0.350	-0.345	**-0.354**	-0.327	-0.295
Systolic BP	0.189	-0.002^3^	0.205	0.175	**0.218**	0.168	0.138
Diastolic BP	0.220	0.012^3^	**0.230**	0.198	**0.228**	0.187	0.168
HbA1c^2^	0.178	-0.044^3^	0.218	0.249	**0.261**	0.214	0.123
HOMA-IR^2^	0.497	-0.005^3^	0.557	0.570	**0.572**	0.517	0.362
Glucose^2^	0.187	-0.093	0.254	0.260	**0.267**	0.219	0.122
FEMALES							
Triglycerides^2^	0.306	-0.033^3^	0.326	0.342	**0.404**	0.281	0.205
HDL-C	-0.283	0.074	-0.314	-0.301	**-0.364**	-0.265	-0.172
Systolic BP	0.148	-0.030^3^	**0.163**	0.135	0.161	0.126	0.078
Diastolic BP	0.172	-0.019^3^	**0.186**	0.136	0.170	0.149	0.081
HbA1c^2^	0.202	-0.029^3^	0.220	0.208	**0.256**	0.177	0.103
HOMA-IR^2^	0.516	-0.052^3^	0.550	0.493	**0.574**	0.462	0.288
Glucose^2^	0.281	-0.017^3^	0.298	0.303	**0.347**	0.268	0.183

^1^Adjusted for age.

^2^nLog transformed.

All correlation coefficients are significant (P<0.05) except: ^3^P>0.05. The index associated with the highest correlative strength to the variable in the same row is highlighted.

### Associations between obesity measures, cardiometabolic risk features and type 2 diabetes

**Fig 1:**
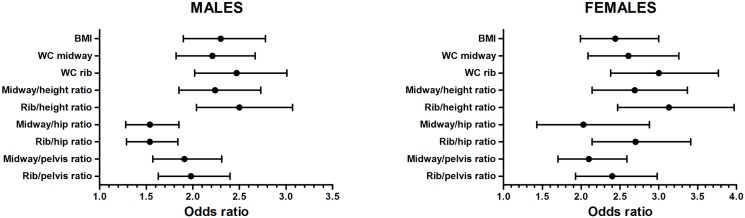
Odds ratios (95% CI) of having three or more cardiometabolic risk features for a one standard deviation increase in each obesity measure. Results are stratified by gender and adjusted for age. All models exclude subjects with type 2 diabetes.

**Fig 2:**
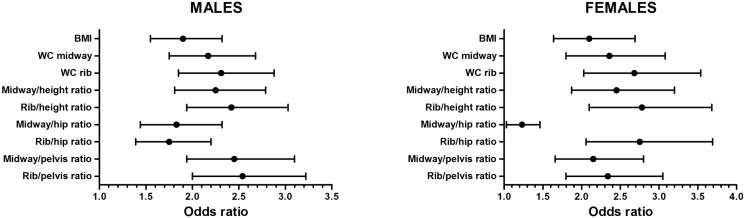
Odds ratios (95% CI) of having type 2 diabetes for a one standard deviation increase in each obesity measure. Results are stratified by gender and adjusted for age.

The results from regression models examining adiposity variable associations with individual metabolic risk factors (S1 Fig), three or more cardiometabolic risk features (Fig 1) and type 2 diabetes (Fig 2) are shown. Results are adjusted for age and odds ratios represent the odds associated with a one standard deviation increase in each obesity measure. Although the strength of relationship varied according index type, WC rib or rib-derived indices displayed, without exception, stronger associations with individual cardiometabolic risk factors, metabolic feature clustering and type 2 diabetes, in both genders. In general, stronger relationships with cardiometabolic variables were noted in women, with differences between BMI and central obesity being less pronounced in male subjects.

### Receiver operating characteristic curve analysis

**Fig 3:**
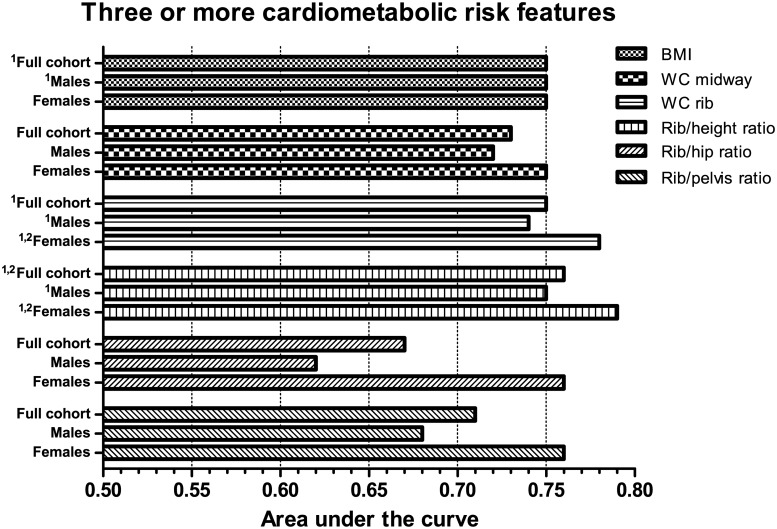
Adjusted area under the receiver operating characteristic curve values for selected obesity measures to discriminate subjects with three or more cardiometabolic risk features. Bars represent AUC values. All models exclude subjects with type 2 diabetes. Statistical differences in the AUC values are shown in superscript Arabic numbers as: ^1^P<0.05 compared to WC midway; ^2^P<0.05 compared to BMI.

**Fig 4:**
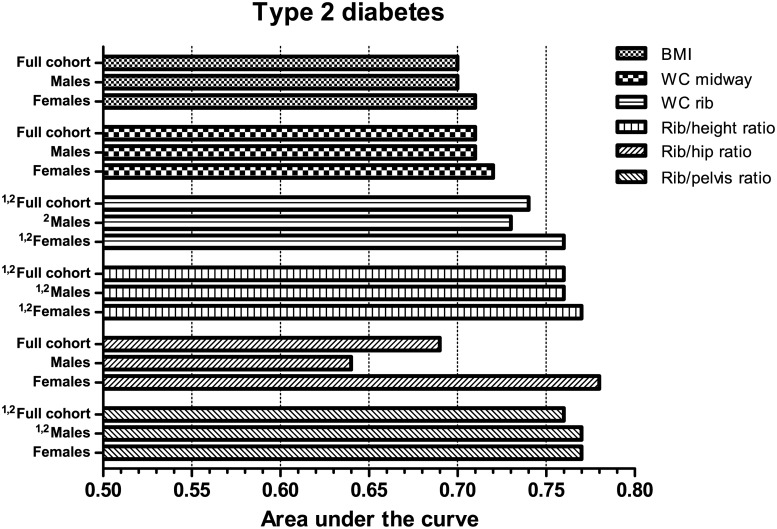
Adjusted area under the receiver operating characteristic curve values for selected obesity measures to discriminate subjects with type 2 diabetes. Bars represent AUC values. Statistical differences in the AUC values are shown in superscript Arabic numbers as: ^1^P<0.05 compared to WC midway; ^2^P<0.05 compared to BMI.

**Fig 5:**
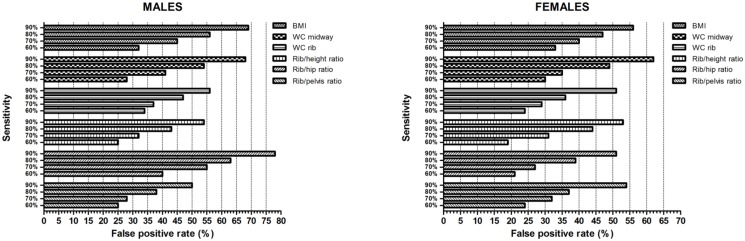
False positive rates corresponding to 90%, 80%, 70% and 60% sensitivities for selected obesity measures to classify subjects with type 2 diabetes. Results are stratified by gender and adjusted for age. Bars represent false positive rates (percentages).

In ROC analysis, both WC rib and Rib/height ratio demonstrated a significantly higher AUC to detect three or more cardiometabolic risk features compared to WC midway in male subjects (Fig 3). In females, significant differences in the AUC were observed when compared to both WC midway and BMI. For type 2 diabetes (Fig 4), WC rib measures showed a higher discriminatory capacity in both genders, with the exception of the Rib/hip ratio in men. Rib-derived indices improved discrimination by 3–7% compared to BMI and 2–6% compared to WC midway (in men) and 5–7% compared to BMI and 4–6% compared to WC midway (in women). Rib measures also displayed greater specificity across a range of sensitivities (Fig 5). At higher sensitivities classification accuracy was improved by as much as 10% or more. However, false positive rates for the Rib/hip ratio were noticeably increased when compared to other adiposity variables in men.

### Evaluation of prediction models

**Fig 6:**
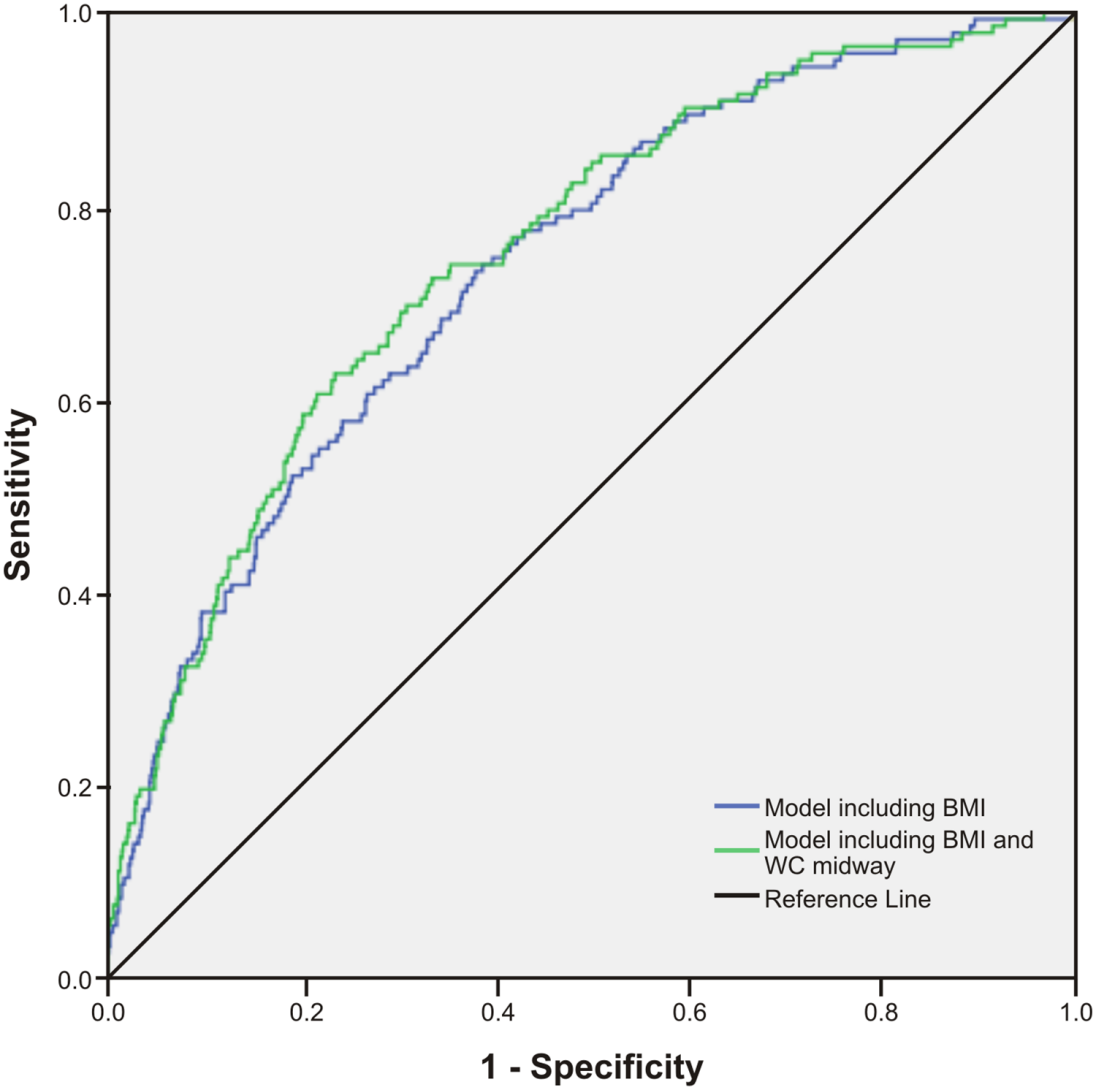
Receiver operating characteristic curves for prediction models to discriminate subjects with type 2 diabetes. Figures show ROC curves for a model including BMI and a model including BMI and WC midway. All models include age and gender.

**Fig 7:**
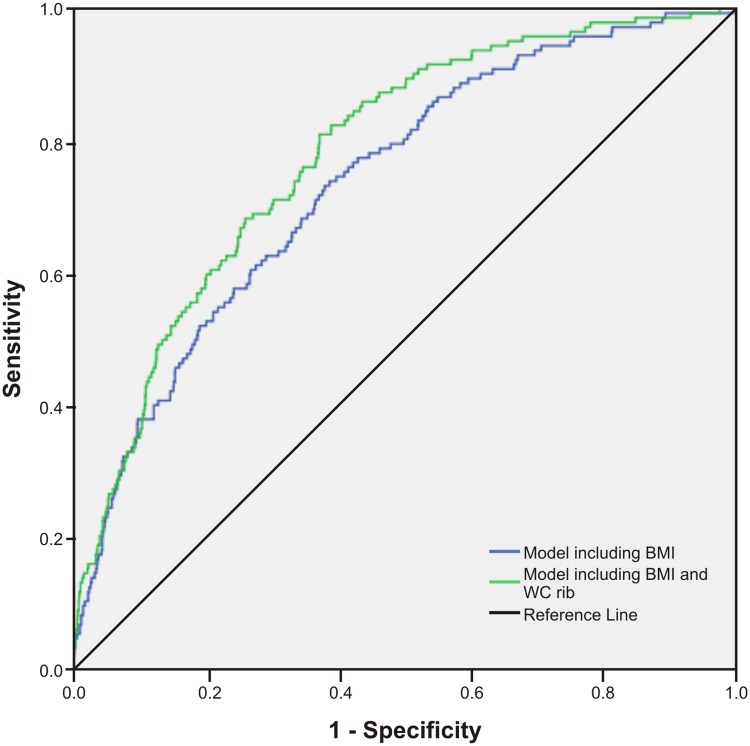
Receiver operating characteristic curves for prediction models to discriminate subjects with type 2 diabetes. Figures show ROC curves for a model including BMI and a model including BMI and WC rib. All models include age and gender.

**Fig 8:**
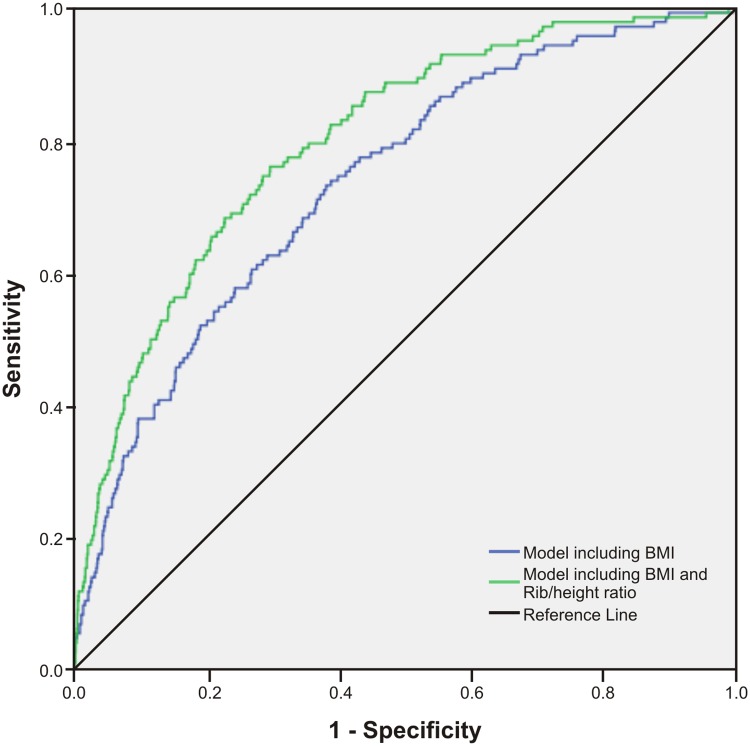
Receiver operating characteristic curves for prediction models to discriminate subjects with type 2 diabetes. Figures show ROC curves for a model including BMI and a model including BMI and Rib/height ratio. All models include age and gender.

**Fig 9:**
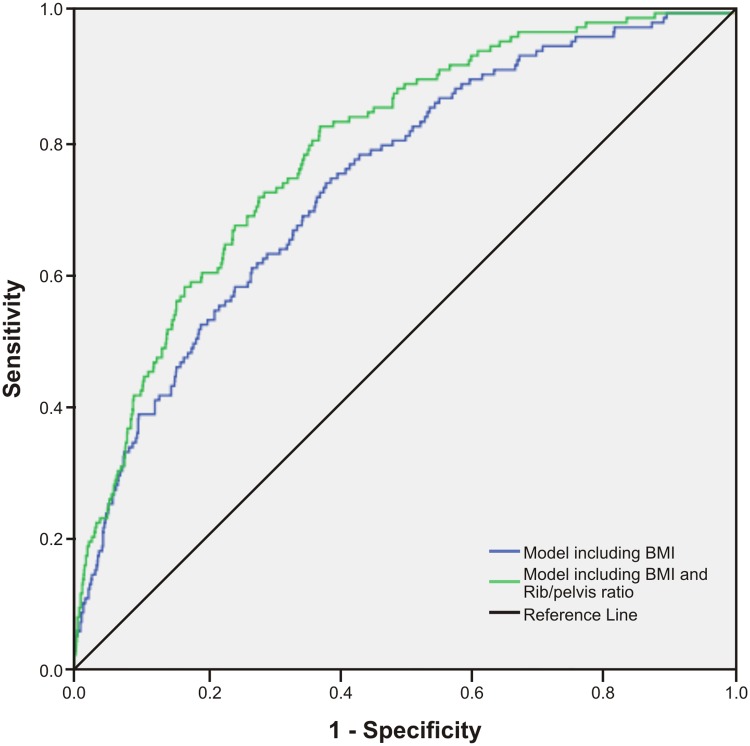
Receiver operating characteristic curves for prediction models to discriminate subjects with type 2 diabetes. Figures show ROC curves for a model including BMI and a model including BMI and Rib/pelvis ratio. All models include age and gender.

As presented in Table 3, we examined prediction models for type 2 diabetes which included BMI and an additional central obesity measure. The HL test showed P values that were non-significant, suggesting that model fits were acceptable. Additionally, the LR chi-squares were reduced in models including central obesity variables, indicating improved goodness-of-fit. Using the IDI statistic, a significant but marginal increase in discrimination was observed for WC midway, with a small and non-significant increase in the AUC (0.75, P = 0.127) (Fig 6). In contrast, a prediction model including BMI and WC rib measures displayed a significantly higher AUC (Figs 7–9) for WC rib (0.78, P = 0.003), Rib/height ratio (0.80, P<0.001) and Rib/pelvis ratio (0.79, P<0.001) when compared to a model with BMI alone (0.74).

**Table 3:** Tests of calibration, goodness-of-fit and discrimination for prediction models to identify subjects with type 2 diabetes.

Model^1^	HL *x* ^2^ (P value)	LR *x* ^2^ (P value)	AUC (95% CI)	IDI (95% CI)
BMI alone	4.39 (0.82)	919.38 (<0.001)	0.74 (0.70–0.78)	-
BMI and WC midway	2.32 (0.97)	900.78 (<0.001)	0.75 (0.71–0.79)^2^	0.0177 (0.002–0.0334)
BMI and WC rib	5.01 (0.76)	877.54 (<0.001)	0.78 (0.74–0.82)^3^	0.0283 (0.0111–0.0455)
BMI and Rib/height ratio	5.34 (0.72)	858.75 (<0.001)	0.80 (0.76–0.84)^4^	0.0364 (0.0162–0.0566)
BMI and Rib/pelvis ratio	6.58 (0.58)	860.73 (<0.001)	0.79 (0.75–0.82)^5^	0.0290 (0.0135–0.0445)

^1^All models include age and gender.

^2^P value = 0.127 compared to model with BMI alone.

^3^P value = 0.003 compared to model with BMI alone.

^4^P value<0.001 compared to model with BMI alone.

^5^P value<0.001 compared to model with BMI alone.

## Discussion

Both the WHO and International Diabetes Federation (IDF) have suggested midway WC measurement as the preferred method for central obesity assessment [12,24]. In contrast, the United States National Institutes of Health (NIH) recommends measuring WC at the superior border of the iliac crest [25]. However, there is a lack of scientific rationale to support either of these measurement protocols [26]. Although previous studies have compared these two criteria, to the best of our knowledge, this is the first to comprehensively evaluate rib WC measures and both WC midway and BMI as predictors of cardiometabolic risk and type 2 diabetes. Our findings suggest that WC rib, rather than WC midway, is a better indicator of central obesity as it improves discrimination of type 2 diabetes within our population. One possible explanation for this relationship may be that rib-level measurement is less influenced by inter-individual variables such as body posture or elasticity of the abdominal wall, which are partly unrelated to actual body adiposity.

The results from previous research investigating different WC measurement criteria are conflicting. A systematic review of 120 studies [27] concluded that the measurement procedure had no substantial influence on WC relationships with morbidity and mortality, leading the authors to recommend the NIH protocol as it may be more readily adapted by health practitioners and is more suitable for self-measurement by the general public. However, effect sizes and discriminatory differences between WC sites were not compared. In contrast, Ma et al. [28] found WC midway to be slightly better than NIH-recommended iliac measurement to predict hypertension, metabolic syndrome and diabetes. Nevertheless, WC rib was not assessed in this study. Bosy-Westphal et al. [26] also observed lower associations between the iliac site and metabolic characteristics and visceral adipose tissue (VAT) in females. Relationships between cardiometabolic variables and WC midway and rib were similar in men, while WC rib was more strongly correlated with VAT in women.

Regardless of controversies surrounding WC measurement protocol, both advantages and disadvantages exist regarding the general application of central obesity assessment within clinical practice. Although some studies have suggested WC to be the simplest and best overall method for cardiometabolic health appraisal [29], as metabolic risk cut-points for WC are different between genders, and vary between ethnic groups [12,30], the practical usability of WC measurement is still uncertain [11].

In keeping with other findings [13,31], our results imply that transformations of WC may improve discrimination of cardiometabolic outcomes. The use of a ratio to define central obesity is also potentially beneficial as it might allow uniform diagnostic thresholds to be used (between ethnicities, genders or both), making it attractive from a public health perspective [32,33]. Notably, however, the WHR was a markedly inferior discriminator of risk in male subjects within this sample. Reduced associations for WHR were also observed by Schneider et al. [34], who theorised that as both WC and hip circumference exhibit strong relationships with cardiometabolic features, a ratio of the two may show less. Additionally, both measures may increase or decrease proportionally in an individual [35]. It could be that sex differences observed for WHR are due to gender variations in body composition, and that changes in hip circumference, relative to WC, are more pronounced in middle-aged men than in women.

Although WC rib measures demonstrated stronger relationships with metabolic variables, consistent with previous research [11], our study also revealed that anthropometric associations with a majority of cardiometabolic risk factors and type 2 diabetes were reduced in men. One possible explanation for this finding is the greater prevalence of overweight and obesity amongst males within this population, perhaps minimising associations and predictive abilities. It was also noted that discriminatory differences between central obesity and BMI were greater when detecting type 2 diabetes compared to a clustering of metabolic variables, in both genders. A reason for this may be that central adiposity independently predicts type 2 diabetes, beyond traditionally assessed cardiometabolic disease markers [36].

Compared with BMI, central obesity is thought to be more strongly correlated with VAT [10]. Research has implied that fatty acids released from VAT drain into the liver and skeletal muscle causing metabolic dysfunction within these organs [37]. Adipokines secreted from VAT may also contribute to cardiometabolic disease through inflammation of vascular tissue [9]. Increased VAT has been shown to be associated with increased risk of dyslipidaemia, hypertension and type 2 diabetes [38,39]. Consequently, observed differences in discrimination for cardiometabolic disease mediators and type 2 diabetes suggest that central obesity should be independently evaluated as a cardiometabolic risk factor, and that its inclusion as a mandatory component of the metabolic syndrome may be appropriate [24].

However, the findings from previous studies which have contrasted central obesity (either WC, WHtR or WHR) with BMI to discriminate cardiometabolic conditions have been inconclusive [11,15]. Possible reasons for variations between studies may include different WC measurement protocols or dissimilar methods for classifying cardiometabolic outcomes. Although AUC values for central obesity measures are frequently reported to be larger when compared to BMI for predicting type 2 diabetes [40], as the AUC lacks clinical relevance, there is argument against using it as a summary statistic of the ROC curve as similar AUC values may have different diagnostic properties [21]. Though other studies have reported metabolic risk thresholds for obesity indices based on maximum sensitivity, optimal sensitivity and specificity or the shortest distance to the y axis [12], cut-points are necessarily arbitrary, and may vary between populations.

Central obesity measures have been proposed as stand-alone, pre-screening tools [33] for use in high-risk populations to enable clinicians to detect those who might benefit from further diagnostic or therapeutic procedures [41,42]. In this scenario it is desirable to optimise sensitivity (the percentage of people with or at risk of a condition, who would be correctly identified), in order to rule out healthy subjects. Importantly, by comparing false positive rates (the proportion of healthy individuals who would be misclassified) across a range of sensitivities for multiple indices, our results demonstrate WC rib measures to be more accurate classifiers, at higher sensitivities, compared to WC midway and BMI.

Nevertheless, debate exists regarding the clinical efficacy of central obesity measurement. To some extent this is due to a lack of evidence regarding how much of an increase in predictive accuracy central obesity measures might add over traditionally assessed cardiometabolic risk indicators [11]. Though our findings suggest that central obesity variables may provide additional prognostic information, these results also indicate that the degree of improvement is significantly influenced by measurement procedure.

While only requiring a flexible measuring tape, midway WC is difficult to obtain as it requires the identification of two bony landmarks, a computed distance between the two, and a circumference evaluation—essentially four separate measurements. As central obesity assessment competes for the limited time available during patient appraisal, and necessitates specific training to ensure reliable data are obtained [10], a simpler measurement protocol is desirable. WC rib is more easily determined and offers a more practical method for use within healthcare practice and epidemiological research, and would be equally suitable for self-assessment. Furthermore, Bosy-Westphal et al. [26] and Wang et al. [43] also concluded that WC rib had a higher reproducibility. As measurement error may limit the minimal detectable difference in a parameter [26], it is possible that the higher discriminatory accuracy we observed may be due to greater measurement precision.

Though our findings are of potential public health and clinical significance, several limitations should be considered. Given the modest number of outcomes within our sample we did not adjust for multiple factors in analyses. Our primary aim was to compare general and central obesity relationships, rather than to determine overall strengths of association. Nevertheless, the possibility that confounding features may influence adiposity variables in different ways cannot be discounted and future studies with larger samples might find different relationships. Also, as cross-sectional data precludes examination of the temporal relationship between obesity measures and cardiometabolic disease, our results may suggest associations, but they do not demonstrate an ability to predict type 2 diabetes.

Equally of concern is that we did not have other WC measurement sites to contrast and that our data were derived from a single primary care based sample. However, Ireland represents a generally ethnically homogeneous population [44]. Consequently, random sampling of subjects and the use of validated methods for data collection ensured internal sample validity and the relationships described may be generalisable to a similar middle-aged, Caucasian-European population. Nonetheless, future studies utilising longitudinal data in different samples will be needed to evaluate the validity and reliability of alternative WC measurements. In particular, it will be necessary to determine whether risk stratification, using central obesity, is clinically useful and superior to currently recommended BMI classification [45].

## Conclusions

In summary, our results indicate that measurement protocol for WC may be important for determining central obesity and assessing cardiometabolic health. Rib-level measures were more strongly related to cardiometabolic risk factors and demonstrated improved discrimination of type 2 diabetes. In light of the increasing prevalence of obesity and cardiometabolic disease worldwide, effective methods that help assess the probability of diabetes development are needed [46,47]. The clinical utility of WC measured at the lowest rib, rib-derived indices or alternative WC measurements as potentially more accurate predictors of metabolic risk and type 2 diabetes, compared to WHO and IDF-recommended WC midway measurement or BMI, deserves further investigation.

## Supporting Information

S1 Figs Odds ratios (95% CI) of having non-optimal cardiometabolic risk features for a one standard deviation increase in each obesity measure. Results are stratified by gender and adjusted for age. Figures show odds ratios (95% CI) regarding obesity measurement associations with high triglycerides, low HDL-C, high blood pressure, insulin resistance and impaired fasting glucose. Models examining impaired fasting glucose exclude subjects with type 2 diabetes.
(PDF)
