# Supplementary material for: Optimal Central Obesity Measurement Site for Assessing Cardiometabolic and Type 2 Diabetes Risk in Middle-Aged Adults
Source: PLoS One. 2015 Jun 4;10(6):e0129088. doi: 10.1371/journal.pone.0129088 (PMC4456242; doi:10.1371/journal.pone.0129088)

S1 Figs. Odds ratios (95% CI) of having non-optimal cardiometabolic risk features for a one standard deviation increase in each obesity measure.

HIGH TRIGLYCERIDES

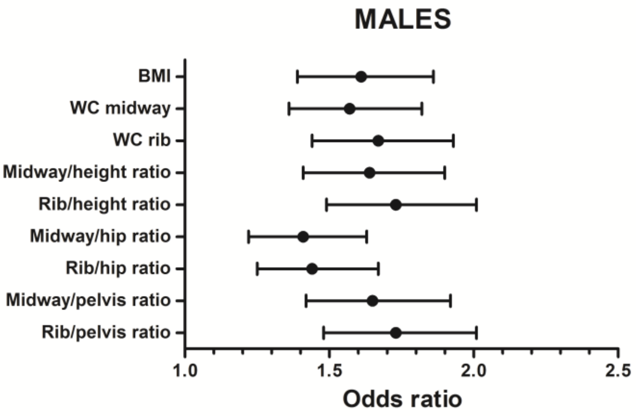

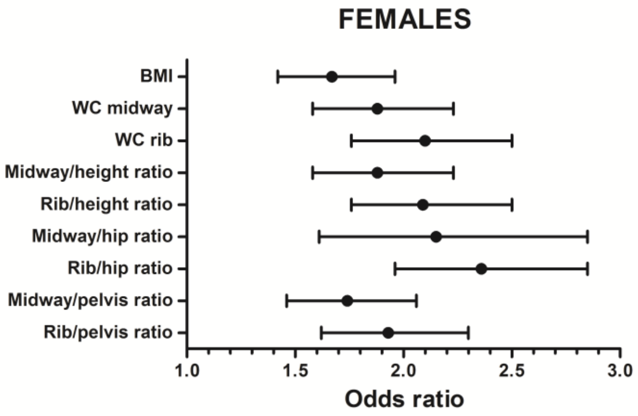

LOW HDL-C

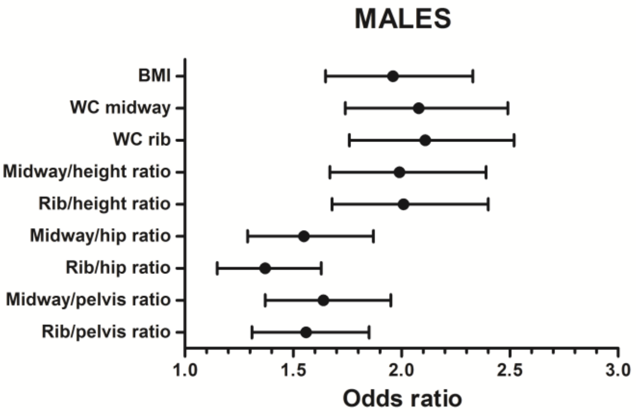

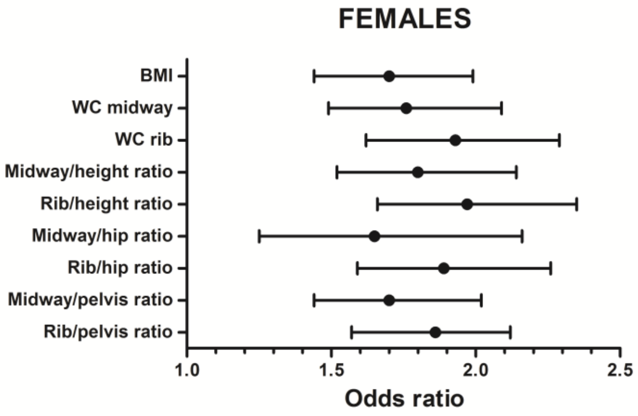

HIGH BLOOD PRESSURE

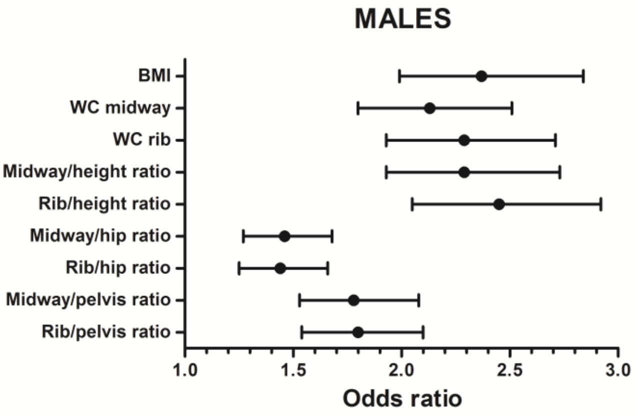

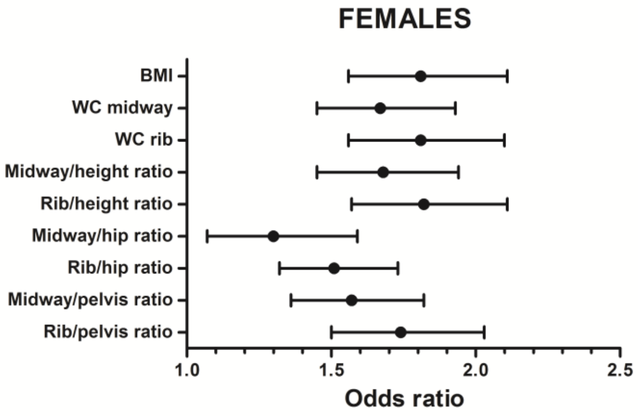

INSULIN RESISTANCE

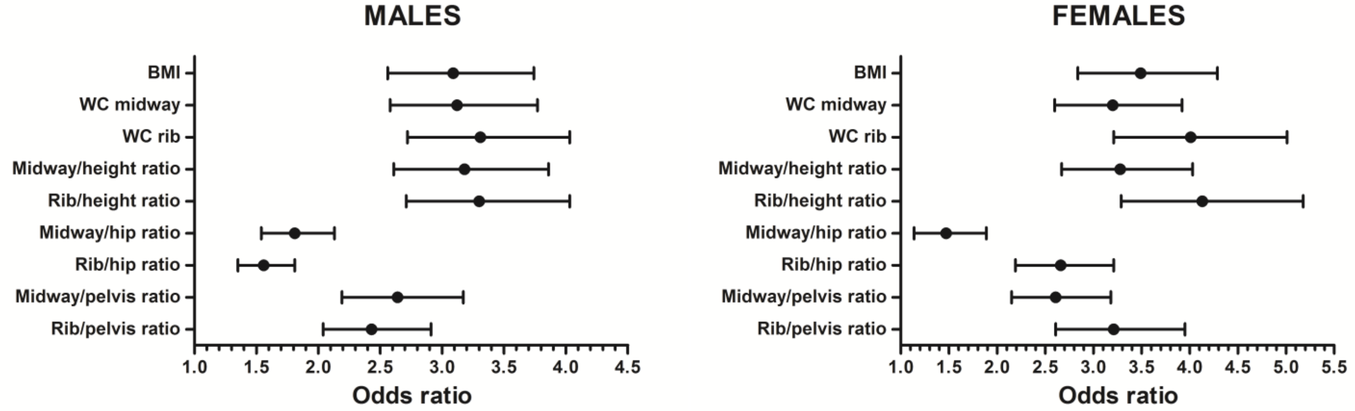

IMPAIRED FASTING GLUCOSE

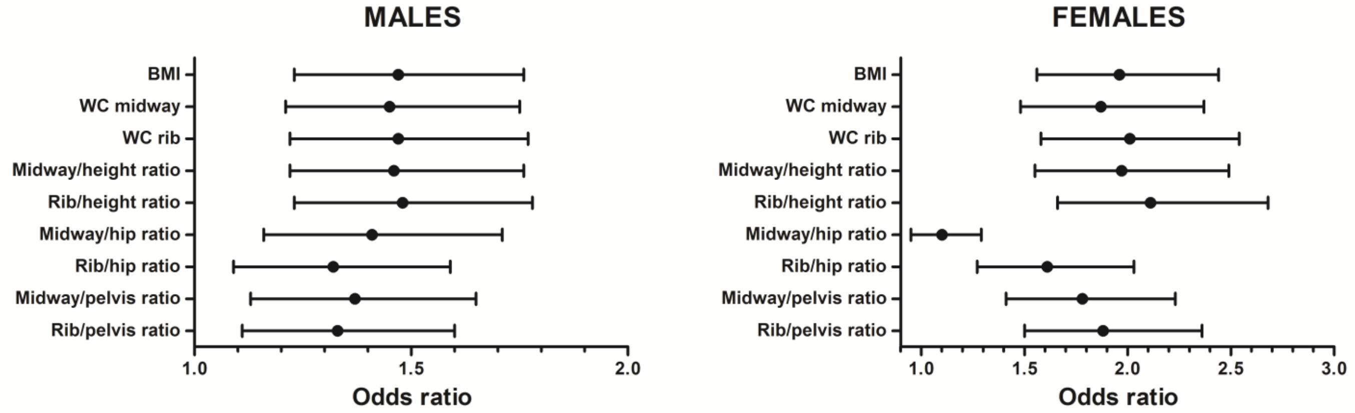

Supplement: S1 Figs — Results are stratified by gender and adjusted for age. Figures show odds ratios (95% CI) regarding obesity measurement associations with high triglycerides, low HDL-C, high blood pressure, insulin resistance and impaired fasting glucose. Models examining impaired fasting glucose exclude subjects with type 2 diabetes. (PDF) [file pone.0129088.s001.pdf]
